# Association between female reproductive factors and intraocular pressure according to glaucoma status: A cross-sectional study of the Korea National Health and Nutrition Examination Survey

**DOI:** 10.1371/journal.pone.0353666

**Published:** 2026-07-29

**Authors:** Ji Eun Song, Mi Yeon Lee, Chungkwon Yoo, Joon Mo Kim

**Affiliations:** 1 Department of Ophthalmology, Kangwon National University Hospital, Kangwon National University School of Medicine, Chuncheon, Republic of Korea; 2 Division of Biostatistics, Department of Academic Research, Kangbuk Samsung Hospital, Seoul, Republic of Korea; 3 Department of Ophthalmology, Korea University Medical Center, Korea University College of Medicine, Seoul, Republic of Korea; 4 Department of Ophthalmology, Kangbuk Samsung Hospital, Sungkyunkwan University School of Medicine, Seoul, Republic of Korea; Seirei Hamamatsu General Hospital, JAPAN

## Abstract

**Background:**

To investigate the association between endogenous estrogen exposure and intraocular pressure (IOP) in postmenopausal women stratified by glaucoma status.

**Methods:**

This population-based, cross-sectional study analyzed 1,823 postmenopausal women aged ≥50 years from the Korea National Health and Nutrition Examination Survey (2010–2011). Age at menarche, and menopause, reproductive span, and time since menopause were used as surrogate markers of lifetime estrogen exposure. Multivariate linear regression analyses were performed for the non-glaucoma and glaucoma groups, adjusting for confounders.

**Results:**

Among women without glaucoma, indicators of longer estrogen exposure were significantly associated with higher IOP. Menopause at the age of ≥49 years and reproductive span of ≥33 years were associated with IOP increases of 0.79 mmHg and 0.88 mmHg, respectively. Conversely, in women with glaucoma, longer estimated estrogen exposure was associated with lower IOP. Menopause at ≥48 years of age and reproductive span at ≥31 years were not associated with IOP reduction. These findings demonstrate distinct associations between endogenous estrogen exposure proxies and IOP in women with and without glaucoma.

**Conclusions:**

Our results suggest a context-dependent role of estrogen in ocular physiology, with potential implications for glaucoma risk assessment and the development of preventive strategies in aging women.

## Introduction

Sex-related differences in the prevalence of glaucoma and ocular hypertension have prompted increasing interest in the role of estrogen in the regulation of intraocular pressure (IOP) and optic nerve integrity [1,2]. Although glaucoma affects both sexes, women experience distinct hormonal fluctuations across the lifespan—including menarche, pregnancy, parturition, or menopause—that may influence ocular physiology [1,3]. Endogenous estrogen is increasingly recognized as a pleiotropic hormone whose effects that extend well beyond the reproductive system [4], contributing to the maintenance and functional integrity of multiple organs and tissues in women. Its effects encompass physiological processes, including the maintenance of healthy blood flow [5,6], facilitation of neural activation [7], and regulation of mitochondrial function [8,9], all of which are crucial for cellular health and resilience.

Estrogen has been proposed to reduce IOP and confer neuroprotective effects [10–12]. Consistent with this, clinical observations indicate that postmenopausal women often exhibit a slightly higher IOP and accelerated nerve fiber layer thinning compared with premenopausal women [1,10]. Epidemiological studies have further associated early menopause—which reflects a shorter duration of estrogen exposure—with an increased risk of open-angle glaucoma [11–13]. Conversely, other studies suggest that later menopause and a prolonged reproductive lifespan may be associated with higher IOP or an elevated risk of glaucoma, highlighting the lack of consensus in the current literature [14,15]. These conflicting findings underscore the need to clarify the relationship between cumulative endogenous estrogen exposure and IOP, with reproductive factors serving as surrogate markers.

Lifetime estrogen exposure can be approximated using reproductive factors, including age at menarche, age at menopause, and the interval between them, while recognizing that pregnancy and exogenous hormone use also contribute [2,11]. Population-based surveys generally capture menarche and menopausal ages reliably, making them useful proxies for cumulative endogenous estrogen. As elevated IOP represents the primary modifiable risk factor for glaucoma, elucidating the relationship between reproductive hormone history and IOP may provide insight into sex-specific mechanisms underlying glaucoma [3].

This study conducted a population-based analysis using Korea National Health and Nutrition Examination Survey (KNHANES) data to investigate the associations among female reproductive factors, IOP, and glaucoma pathophysiology. Age at menarche, age at menopause, and reproductive span (the interval between menarche and menopause) were evaluated for independent associations with IOP measured by Goldmann tonometry and for differences according to glaucoma status. By stratifying participants into glaucoma and non-glaucoma groups, this study aimed to determine whether long-term estrogen exposure exerts protective, neutral, or adverse effects on IOP and provide insight into the role of estrogen in glaucoma risk.

## Methods

This study was conducted in accordance with the tenets of the Declaration of Helsinki. The KNHANES was approved by the Institutional Review Board of the Korea Centers for Disease Control and Prevention, and all participants provided written informed consent. Because the KNHANES data are fully de-identified and publicly accessible, this secondary analysis was exempted from further review by the Institutional Review Board of Kangbuk Samsung Hospital (KBSMC 2023-01-026).

### Study design and population

The KNHANES 2010–2011 is a nationwide, cross-sectional survey of the non-institutionalized population of South Korea, utilizing a stratified, multistage, probability-cluster sampling design with rolling enrollment. Among the 17,476 participants, 5,032 women aged ≥19 years who completed the health examination and ophthalmologic assessment were included in the analysis. Conversely, participants with a history of ocular surgery (including cataract, retinal, corneal, or refractive procedures), any ocular or systemic disease affecting vision, prior use of glaucoma medications, and incomplete data on reproductive history or ophthalmic measurements were excluded.

### Interview

Trained research assistants conducted in-home interviews to obtain self-reported data on age at menarche and menopause. Natural menopause was defined as the participant’s reported age at the last menstrual period. For women reporting menopause following gynecologic surgery or radiation therapy and those reporting any other operations before the age of 50 years, which may have induced menopause, the exact date and type of procedure were verified using general practitioner records, including correspondence from medical specialists. Reproductive span was calculated as the interval (years) between menarche and menopause. Participants with artificial menopause and/or bilateral oophorectomy were included in the primary analysis. To assess the robustness of our findings, sensitivity analyses were performed after excluding participants with artificial menopause or bilateral oophorectomy.

### Ophthalmological examination

Ophthalmological examinations were performed by an ophthalmologist affiliated with the Korean Ophthalmological Society (KOS), who received periodic training from the KOS National Epidemiologic Survey Committee. Following an ophthalmology-focused interview, visual acuity was measured using the LogMAR scale. s Refractive status was measured using an automatic refractometry (KR-8800; Topcon, Tokyo, Japan), and IOP was measured using a Goldmann applanation tonometer (GAT; Haag-Streit model BQ-900; Haag-Streit AG, Koeniz, Switzerland) as part of the KNHANES ophthalmological examination. Although IOP was measured in both eyes according to the KNHANES ophthalmological examination protocol, the right-eye IOP was consistently used for the present analysis in both participants without glaucoma and those with glaucoma. Participants with missing or outlier right-eye IOP values were excluded. Slit-lamp examination included assessment of peripheral anterior chamber depth using the Van Herick method (Haag-Streit model BQ-900; Haag-Streit AG, Koeniz, Switzerland). An anterior chamber depth greater than one-fourth of the peripheral corneal thickness was classified as an open angle. Retinal examinations were performed using non-mydriatic fundus photography of both eyes. Images were captured with a digital fundus camera (TRCNW6S; Topcon, Tokyo, Japan). Visual field testing was performed using frequency doubling technology (FDT) (Humphrey Matrix; Carl Zeiss Meditec, Inc., Dublin, CA, USA) with the N-30–1 screening protocol in participants exhibiting elevated IOP (≥22 mm Hg) or glaucomatous optic disc features. Glaucomatous optic discs were determined by any of the following criteria: (1) horizontal or vertical cup-to-disc ratio ≥0.5, (2) nonadherence to the ISNT rule (neuroretinal rim thickness by quadrant: inferior > superior > nasal > temporal), (3) presence of optic disc hemorrhage, or (4) presence of a retinal nerve fiber layer (RNFL) defect. FDT results were considered unreliable and repeated if either the false-positive rate or fixation error rate exceeded 30%.

### Definitions of Open-Angle Glaucoma and control groups

Open‐angle glaucoma (OAG) was diagnosed according to the International Society of Geographical and Epidemiological Ophthalmology (ISGEO) criteria and the established KNHANES protocols. [16,17] Eyes were classified as OAG if they had an open anterior chamber angle—defined as a peripheral anterior chamber depth exceeding one-quarter of the corneal thickness using the Van Herick method—and met either Category I or II diagnostic thresholds. Category I applied when FDT perimetry demonstrated fixation and false‐positive error rates of ≤1. In such cases, glaucoma was confirmed by the presence of any one of the following factors: neuroretinal rim thinning with a horizontal or vertical cup‐to‐disc ratio of ≥0.7 (or an inter‐eye asymmetry ≥0.2, both corresponding to the ≥ 97.5th percentile of the normal KNHANES cohort), optic disc hemorrhage, or an RNFL defect. Additionally, abnormal FDT results were required (defined as at least one localized sensitivity loss consistent with disc appearance or an RNFL defect). Category II criteria were applied when FDT was unavailable or exhibited fixation or false‐positive error rates of ≥2. Diagnosis in these patients relied on either rim thinning with a vertical cup‐to‐disc ratio of ≥0.9 (or asymmetry ≥0.3) or an RNFL defect corresponding to the optic disc appearance.

Control participants were defined as those whose eyes exhibited an intraocular pressure of ≤21 mmHg, maintained open angles on Van Herick assessment, demonstrated non-glaucomatous optic discs with a cup-to-disc ratio of <0.7 and an inter-eye difference of <0.2, showed no RNFL defects or disc hemorrhages, and preserved the ISNT rule.

All initial gradings were conducted by trained ophthalmologists, followed by detailed regrading by a separate, blinded panel of glaucoma specialists. Any discrepancies were adjudicated by a third independent group of specialists.

### Statistical analysis

All analyses were performed using STATA 16.1 (StataCorp, College Station, TX, USA), accounting for the survey’s complex sampling design. Sampling strata, primary sampling units, and sample weights were incorporated to generate population-representative estimates and standard errors (SEs) using Taylor series linearization. Continuous variables were expressed as weighted means ± SE, whereas categorical variables were expressed as weighted proportions (with SE).

Baseline demographic and clinical characteristics were compared between the groups using Pearson’s chi-square test for categorical variables and survey-adjusted general linear models (GLMs) for continuous variables. Because KNHANES uses a complex, stratified, multistage probability sampling design, all analyses were performed using survey procedures that incorporated the sampling weights, stratification variables, and cluster variables provided by KNHANES. Participants with missing or outlier values for key eligibility variables, including right-eye IOP, were excluded according to the predefined exclusion criteria. For other variables, missing values were treated as valid missing values and excluded from the corresponding analyses; no imputation was performed. To preserve the complex survey design, analyses were conducted using subpopulation/domain analysis rather than by physically deleting observations outside the analytic subpopulation. Survey-adjusted GLMs were subsequently used to examine the associations between reproductive factors and IOP. In these models, IOP was treated as a continuous dependent variable, and each reproductive factor was entered as an independent variable. Survey-adjusted GLMs were subsequently used to examine the associations between reproductive factors and intraocular pressure (IOP). In these models, IOP was treated as a continuous dependent variable, and each reproductive factor was entered as an independent variable. Three sequential models were constructed. Model 1 was adjusted for age. Model 2 was adjusted for age, diabetes mellitus, and systolic and diastolic blood pressure. Model 3 was adjusted for age, diabetes mellitus, systolic and diastolic blood pressure, body mass index (BMI), triglycerides, and low-density lipoprotein cholesterol levels. Covariates were selected based on clinical relevance, previous literature, and baseline differences between groups. The results are presented as β coefficients with 95% confidence intervals. The results of the survey-adjusted GLMs are presented as β coefficients with 95% confidence intervals (CIs). Covariates were selected based on baseline differences (p < 0.05) and included age, smoking status, diabetic mellitus, systemic hypertension, BMI, triglycerides, and total cholesterol and low-density lipoprotein levels. All tests were two-sided, and a p < 0.05 was considered significant.

Logistic regression analyses were conducted to calculate odds ratios (ORs) and their 95% confidence intervals (CIs), along with β‐coefficients. The associations between reproductive factors and elevated IOP were expressed as ORs with 95% CIs. All statistical tests were two-sided, and p-values <0.05 were considered statistically significant.

## Results

### Participant characteristics

A total of 1,823 women were included in the analysis, of whom 111 (6.1%) met the ISGEO criteria for open-angle glaucoma. Compared with participants with non-glaucoma, those with glaucoma were older (62.3 ± 1.3 vs 58.9 ± 0.3 years, P = 0.010), had older age at menarche (16.27 ± 0.20 vs 15.80 ± 0.07 years, P = 0.025), higher systolic blood pressure (127.9 ± 1.7 vs 124.3 ± 0.6 mmHg, P = 0.043), higher prevalence of hypertension (46.8% vs 41.8%, P = 0.033), longer duration of menarche until the study (47.9 ± 9.1 vs 44.34 ± 8.18 years, P = 0.021), and shorter duration after menopause (10.37 ± 0.29 vs 11.41 ± 9.4 years, P = 0.031). No significant differences were observed between groups in BMI (24.96 ± 0.53 vs 24.33 ± 0.09 kg/m^2^), diastolic blood pressure (79.51 ± 1.23 vs 77.08 ± 0.35 mmHg), fasting glucose level (99.19 ± 1.98 vs 98.68 ± 0.58 mg/dL), lipid profiles, age at menopause (48.91 ± 0.58 vs 48.66 ± 0.15 years), reproductive span (32.64 ± 0.62 vs 32.88 ± 0.16 years), and hormone replacement therapy use (14.4 ± 0.4% vs 16.6 ± 1.1%) (Table 1). Among the 1,823 participants, 276 reported artificial menopause; 13 were classified as having glaucoma and 263 as not having glaucoma. In addition, 79 participants reported a history of bilateral oophorectomy; among them, 3 had glaucoma and 76 did not.

**Table 1:** Characteristics of participants according to glaucoma status.

	Glaucoma (n = 111, 6.1%)		Non-glaucoma (n = 1,712, 93.9%)		*P* value
	Mean (SE)	95% CI	Mean (SE)	95% CI	
Age (years)	62.33 (1.32)	(59.73-64.94)	58.94 (0.27)	(58.41-59.46)	**0.01**
BMI (kg/m^2^)	24.96 (0.53)	(23.93-26)	24.33 (0.09)	(24.15-24.5)	0.229
Waist circumference (cm)	84.03 (1.28)	(81.53-86.54)	82.09 (0.28)	(81.55-82.64)	0.134
Systolic blood pressure (mmHg)	127.9 (1.68)	(124.6-131.2)	124.31 (0.6)	(123.13-125.5)	**0.043**
Diastolic blood pressure (mmHg)	79.51 (1.23)	(77.08-81.93)	77.08 (0.35)	(76.39-77.77)	0.068
Glucose (mg/dL)	99.19 (1.98)	(95.29-103.09)	98.68 (0.58)	(97.54-99.82)	0.801
Total cholesterol (mg/dL)	203.76 (4.44)	(195.03-212.49)	201.42 (1.12)	(199.22-203.62)	0.613
HDL-C (mg/dL)	52.09 (1.23)	(49.67-54.51)	53.7 (0.37)	(52.97-54.44)	0.200
LDL-C (mg/dL)	126.3 (8.54)	(109.51-143.1)	126.02 (1.96)	(122.17-129.88)	0.975
Triglycerides (mg/dL)	146.85 (8.59)	(129.96-163.75)	132.3 (2.14)	(128.09-136.5)	0.101
Diabetes					0.802
Diabetes	14.5 (4.1)	(8.09-24.48)	11.9 (0.9)	(10.23-13.83)	
Pre DM	19.0 (4.7)	(11.32-30.03)	20.5 (1.2)	(18.16-23.02)	
Hypertension					**0.033**
Hypertension	46.8 (5.7)	(36.05-57.92)	41.8 (1.4)	(39.07-44.65)	
Pre-hypertension	29.7 (5.3)	(20.41-40.91)	21.1 (1.2)	(18.81-23.68)	
IOP (mmHg)	14.27 (0.38)	(13.69-14.08)	13.88 (0.10)	(13.53-15.01)	0.312
Age at menarche (years)	16.27 (0.2)	(15.87-16.67)	15.8 (0.07)	(15.67-15.94)	**0.025**
Age at menopause (years)	48.91 (0.58)	(47.77-50.06)	48.66 (0.15)	(48.36-48.97)	0.674
Interval from menarche to menopause (years)	32.64 (0.62)	(31.41-33.87)	32.88 (0.16)	(32.56-33.2)	0.711
Duration of menarche until the study	47.9 (9.1)	(29-74)	44.34 (8.18)	(15-70)	**0.021**
Duration after menopause (years)	10.37 (0.29)	(9.79-10.94)	11.41 (9.4)	(0-48)	0.013
Hormone replacement Therapy use (%)	14.4 (0.4)	(7.7-25.34)	16.6 (1.1)	(14.59-18.92)	0.645

Pearson’s chi-square test was analyzed using categorical variables, whereas general linear models were analyzed using continuous variables.

SE, standard error; CI, confidence interval; BMI, body mass index; DM, diabetes mellitus; HDL-C, high-density lipoprotein cholesterol; LDL-C, low-density lipoprotein cholesterol.

### Reproductive factors and IOP in patients without glaucoma

Among the 1,712 women without glaucoma, later menopause and a longer reproductive span were associated with higher mean IOP, whereas longer time since menarche and longer duration after menopause were linked to lower IOP (Table 2). In fully adjusted linear regression (Model 3), women who experienced menopause at ≥49 years of age had a 0.79 mmHg higher IOP (95% CI: 0.16 to 1.41, P = 0.014). Women with a reproductive span of ≥33 years had a 0.88 mmHg higher IOP (95% CI: 0.23 to 1.52, P = 0.008). Conversely, women who were ≥50 years past menarche had a 1.22 mmHg lower IOP (95% CI: −2.17 to −0.26, P = 0.013), and women ≥6 years post-menopause had lower IOP by 0.92 mmHg (95% CI: −1.68 to −0.16, P = 0.017). Age at menarche was not significantly associated with IOP.

**Table 2:** Effect of reproductive factors on the intraocular pressure in non-glaucoma subjects: Age at menarche, age at menopause, interval from menarche to menopause, duration from menarche, and duration after menopause.

Reproductive Factor	Category	Mean IOP (SEM)	Model 1		Model 2		Model 3	
			β (95% CI)	p-value	β (95% CI)	p-value	β (95% CI)	p-value
Age at menarche	< 12	14.76 (0.96)	0 (reference)		0 (reference)		0 (reference)	
	≥ 12	13.87 (0.10)	−0.92 (−2.81-0.98)	0.341	−0.99 (−2.91-0.94)	0.314	−1.73 (−3.64-0.17)	0.074
Age at menopause	< 49	13.75 (0.13)	0 (reference)		0 (reference)		0 (reference)	
	≥ 49	13.97 (0.13)	0.22 (−0.11-0.55)	0.196	0.26 (−0.08-0.6)	0.136	0.79 (0.16-1.41)	**0.014**
Interval from menarche to menopause	< 33	13.71 (0.14)	0 (reference)		0 (reference)		0 (reference)	
	≥ 33	13.99 (0.12)	0.28 (−0.04-0.6)	0.085	0.25 (−0.09−0.58)	0.152	0.88 (0.23-1.52)	**0.008**
Duration of menarche until the study	< 50	13.92 (0.11)	0 (reference)		0 (reference)		0 (reference)	
	≥ 50	13.73 (0.16)	−0.67 (−1.21--0.14)	**0.014**	−0.76 (−1.34--0.17)	**0.011**	−1.22 (−2.17--0.26)	**0.013**
Duration after menopause	< 6	14.05 (0.15)	0 (reference)		0 (reference)		0 (reference)	
	≥ 6	13.78 (0.11)	−0.48 (−0.89--0.07)	**0.022**	−0.47 (−0.91--0.04)	**0.032**	−0.92 (−1.68--0.16)	**0.017**

General linear models.

Model 1: Adjusted for age.

Model 2: Adjusted for age, diabetes mellitus, systolic blood pressure and diastolic blood pressure.

Model 3: Adjusted for age, diabetes mellitus, systolic blood pressure, diastolic blood pressure, body mass index, triglycerides, and low-density lipoprotein cholesterol levels.

CI, confidence interval; IOP, intraocular pressure; SEM, standard error of the mean.

All values represent aggregate estimates from survey-weighted analyses and do not contain individual-level participant data.

### Reproductive factors and IOP in participants with glaucoma

**Fig 1:**
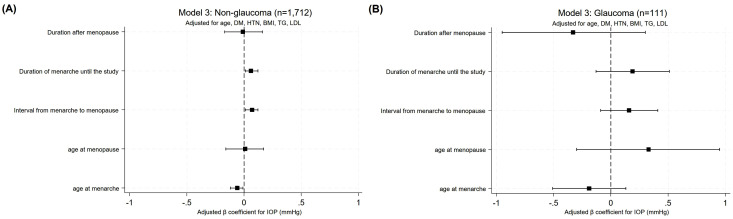
Adjusted associations between reproductive factors and intraocular pressure in participants without and with glaucoma. Forest plots show the fully adjusted β coefficients and 95% confidence intervals in participants without glaucoma (A) and with glaucoma (B). Models were adjusted for age, diabetes mellitus (DM), hypertension (HTN), body mass index (BMI), triglycerides (TG), and low-density lipoprotein cholesterol (LDL-C) levels. The x-axis indicates the adjusted difference in intraocular pressure in mmHg.

In 111 patients with glaucoma, the associations observed for menopausal factors were reversed (Table 3). Later menarche (≥16 years) was associated with a 2.23 mmHg lower IOP (95% CI: −3.91 to −0.55, P = 0.01) in fully adjusted models. Greater years since menarche was also linked to higher IOP (β = 3.62 mmHg, 95% CI: 0.39 to 6.85, P = 0.028), whereas reproductive span, age at menopause and years after menopause showed no consistent associations. The fully adjusted associations between reproductive factors and IOP are visualized in Fig 1.

**Table 3:** Effect of reproductive factors on the intraocular pressure in glaucoma patients: Age at menarche, age at menopause, interval from menarche to menopause, duration from menarche, and duration after menopause.

Reproductive Factor	Category	Mean IOP (SEM)	Model 1		Model 2		Model 3	
			β (95% CI)	p-value	β (95% CI)	p-value	β (95% CI)	p-value
Age at menarche	< 16	14.88 (0.49)	0 (reference)		0 (reference)		0 (reference)	
	≥16	13.94 (0.47)	−0.93 (−2.12-0.25)	0.123	−1.35 (−2.53--0.16)	**0.026**	−2.23 (−3.91-0.55)	**0.01**
Age at menopause	< 48	14.56 (0.69)	0 (reference)		0 (reference)		0 (reference)	
	≥48	14.13 (0.38)	−0.34 (−1.67-0.99)	0.618	0.07 (−1.21-1.35)	0.913	−1.04 (−2.55--0.47)	0.176
Interval from menarche to menopause	< 31	14.48 (0.7)	0 (reference)		0 (reference)		0 (reference)	
	≥31	14.18 (0.38)	−0.11 (−1.4-1.18)	0.866	0.54 (−1.63−1.72)	0.365	−0.29 (−2.13--1.54)	0.752
Duration of menarche until the study	< 42	13.71 (0.54)	0 (reference)		0 (reference)		0 (reference)	
	≥42	14.64 (0.48)	−0.32 (−1.92-1.28)	0.695	−0.45 (−2.13-1.24)	0.602	3.62 (0.39-6.85)	**0.028**
Duration after menopause	< 6	13.75 (0.75)	0 (reference)		0 (reference)		0 (reference)	
	≥6	14.48 (0.44)	−0.58 (−2.49-1.33)	0.549	−0.76 (−2.63-1.11)	0.424	−0.06 (−4.14-4.01)	0.975

General linear models.

Model 1: Adjusted for age.

Model 2: Adjusted for age, diabetes mellitus, systolic blood pressure and diastolic blood pressure.

Model 3: Adjusted for age, diabetes mellitus, systolic blood pressure, diastolic blood pressure, body mass index, triglycerides, and low-density lipoprotein cholesterol levels.

CI, confidence interval; IOP, intraocular pressure; SEM, standard error of the mean.

All values represent aggregate estimates from survey-weighted analyses and do not contain individual-level participant data.

### Sensitivity analysis

After excluding participants with artificial menopause and/or bilateral oophorectomy, the associations between reproductive factors and IOP showed a generally similar pattern to the primary analysis, although the precision of some estimates was reduced because of the smaller sample size (S1–S4 Tables).

## Discussion

Estrogen has been implicated in the maintenance of ocular homeostasis through multiple pathways, including modest effects on IOP and potential influences on optic nerve vulnerability via vascular, mitochondrial, and anti-inflammatory mechanisms [1,18]. Analysis of nationally representative KNHANES 2010–2011 data revealed that the association between endogenous estrogen and IOP is significantly modified by glaucoma status. Among women without glaucoma, indicators of longer lifetime estrogen exposure—specifically, later menopause and an extended reproductive span—were associated with higher IOP, whereas longer postmenopausal duration and older age were associated with lower IOP. These pattern consistent with a previous study in the general Korean population reporting a negative association between age and IOP (coefficient, −0.085; P < 0.001) [19]. Among participants with glaucoma, however, later menopause and a longer reproductive span were associated with 2.10 mmHg and 1.29 mmHg lower IOP, respectively. These findings were comparable in magnitude to previous hormone therapy study, which reported IOP reductions of approximately 0.5–0.6 mmHg [20].

These observations underscore disease-specific hormonal responsiveness, suggesting that the ocular response to estrogen may shift from robust activity in earlier life to reduced receptor sensitivity or altered tissue compliance with aging [21,22]. One of the most notable findings of this study was the differential association between age at menopause and IOP according to glaucoma status. The observation of lower IOP with increased estrogen exposure specifically in glaucoma group may be explained by the antagonistic effects of estrogen on transforming growth factor-beta (TGF-β) signaling. TGF-β2 is a key cytokine that is significantly upregulated in the aqueous humor and trabecular meshwork of patients with glaucoma, promoting excessive extracellular matrix deposition and thereby increasing outflow resistance and IOP [23–25]. Experimental evidence from ocular and non-ocular fibrotic models indicates that estrogen can counteract these TGF-β-induced profibrotic effects by suppressing Smad2/3 phosphorylation and inhibiting the expression of downstream fibrotic markers [26]. In glaucomatous eyes, which are characterized by elevated TGF-β2 activity, estrogen’s anti-fibrotic actions on outflow pathway tissues may be enhanced, providing a mechanistic basis for the modest IOP-lowering effect. Furthermore, estrogen may contribute to lower IOP by modulating the secretory functions of the ciliary body, potentially reducing aqueous humor production [1,21]. Together, these actions—antifibrotic effects on outflow pathways and inhibition of aqueous secretion—may underlie the observed IOP-lowering effect in patients with glaucoma.

In women without glaucoma, where TGF-β-mediated fibrotic activity is minimal, estrogen may modulate IOP through alternative regulatory pathways. Under non-pathological conditions, the vasodilatory and metabolic effects of estrogen on the ciliary body or its influence on aqueous humor production may predominate [10,11]. In this context, estrogen’s role in maintaining or slightly increasing aqueous humor production could explain the modest positive association with IOP observed in this group. These findings indicate that the clinical impact of estrogen on IOP is determined by the underlying ocular molecular environment.

The conventional aqueous humor outflow pathway, which includes the trabecular meshwork and Schlemm’s canal, serves as the primary regulator of IOP homeostasis [27]. Estrogen exerts its effects on these tissues via three receptor subtypes: the classical nuclear receptors ERα and ERβ and the G-protein-coupled estrogen receptor (GPER1) [27,28]. The differential effects of estrogen observed in this study may arise from the context-dependent recruitment of these receptors. Although GPER1 is most abundantly expressed in human TM, its functional role likely shifts from maintaining homeostasis in healthy eyes to acting as a potent anti-fibrotic mediator in glaucomatous eyes. In the pathological environment of OAG, where the TM undergoes structural remodeling, the activation of ERα and GPER1 may be specifically sensitized to reduce outflow resistance by modulating cytoskeletal dynamics [1,29]. In healthy eyes lacking fibrotic stress, estrogen may engage alternative regulatory pathways, as the healthy trabecular meshwork may rely on different homeostatic setpoints. These observations suggest that the clinical effect of estrogen on IOP are shaped by disease-modified responsiveness, in which the structural integrity of the outflow tissues pathways determines the clinical manifestation of lifetime estrogen exposure.

In addition to its role in IOP regulation, the potential neuroprotective effects of estrogen warrant consideration as a complementary factor in understanding the pathophysiology of glaucoma. Estrogen influences vascular tone and blood flow across organs and tissues and exerts neuroprotective effects via non-vascular mechanisms [30,31]. Furthermore, its ability to stabilize mitochondrial function and reduce oxidative stress in optic nerve head astrocytes may confer clinical benefits in glaucomatous eyes, independent of IOP modulation [1,7,25,32]. Although the cross-sectional design of this study precludes definitive causal inferences regarding glaucoma risk, the observed differential associations between estrogen exposure and IOP highlights the complex and multifaceted influence of hormonal status on the ocular environment. These findings suggest that a patient’s reproductive history may be a relevant factor to consider along with traditional clinical parameters, potentially offering deeper insights into the individual variability of glaucoma progression profiles.

This study has some limitations. First, its cross-sectional design precludes the establishment of causal or temporal relationships. Data on age at menarche and menopause were self-reported, which may be subject to recall bias, particularly among those who reached menopause decades earlier. However, recall bias is a common challenge in large-scale epidemiological surveys, and the use of reproductive proxies remains a widely validated method for estimating cumulative estrogen exposure. Second, the study did not differentiate between glaucoma subtypes or severity. The KNHANES 2010–2011 ophthalmological dataset did not include quantitative measures of glaucoma severity, such as standard automated perimetry indices or OCT-derived retinal nerve fiber layer thickness. Therefore, we could not evaluate whether the duration of endogenous estrogen exposure was associated with the severity of glaucoma, including the extent of visual field defects or retinal nerve fiber layer thinning. However, by excluding those currently receiving glaucoma medications, the study population is likely composed of individuals with early-stage or undiagnosed glaucoma. This approach may have strengthened our findings by minimizing the confounding effects of anti-glaucoma medications on IOP, allowing clearer observation of the effect of estrogen. Finally, despite adjusting for multiple systemic covariates, residual confounding factors such as socioeconomic status or other medications potentially correlated with hormone exposure cannot be excluded. Nevertheless, the study provides valuable insights by utilizing a large, nationally representative dataset, supporting high external validity. Future longitudinal studies are warranted to clarify causal relationships and further elucidate the role of reproductive factors in IOP regulation and glaucoma risk.

In conclusion, this study demonstrates a complex, disease-specific association between lifetime endogenous estrogen exposure and IOP in postmenopausal women. Although longer estrogen exposure was linked to higher IOP in women without glaucoma, it tended to correlate with lower IOP in women with glaucoma. These findings suggest that the ocular response to estrogen is context-dependent, being significantly influenced by the underlying physiological or pathological state of the eye. In healthy eyes, the positive association between estrogen and IOP may reflect the hormone’s role in maintaining ocular homeostasis, supporting the optimal functional integrity of the ocular cells and microvasculature. Although the cross-sectional nature of this study limits causal inference, the results underscore the clinical relevance of reproductive history in understanding individual variability in IOP regulation. Further longitudinal investigations are needed to elucidate the mechanisms underlying this disease-modified responsiveness and clarify the role of hormonal factors in glaucoma management.

## Supporting information

S1 Table Association between reproductive factors and intraocular pressure in participants without glaucoma after excluding those with artificial menopause. (DOCX)

S2 Table Association between reproductive factors and intraocular pressure in participants without glaucoma after excluding those with bilateral oophorectomy. (DOCX)

S3 Table Association between reproductive factors and intraocular pressure in participants with glaucoma after excluding those with artificial menopause. (DOCX)

S4 Table Association between reproductive factors and intraocular pressure in participants with glaucoma after excluding those with bilateral oophorectomy. (DOCX)
