# Supplementary material for: Association between female reproductive factors and intraocular pressure according to glaucoma status: A cross-sectional study of the Korea National Health and Nutrition Examination Survey
Source: PLoS One. 2026 Jul 29;21(7):e0353666. doi: 10.1371/journal.pone.0353666 (PMC13419174; doi:10.1371/journal.pone.0353666)
Supplement: S1 Table — (DOCX) [file pone.0353666.s001.docx]

**S1 Table.** Association between reproductive factors and intraocular pressure in participants without glaucoma after excluding those with artificial menopause.

| **Reproductive Factor** | **Category** | **Mean IOP (SEM)** | **Model 1** | | **Model 2** | | **Model 3** | |
| --- | --- | --- | --- | --- | --- | --- | --- | --- |
|  |  |  | **β (95% CI)** | **p-value** | **β (95% CI)** | **p-value** | **β (95% CI)** | **p-value** |
| Age at menarche | < 12 | 15 (0.96) | 0 (reference) |  | 0 (reference) |  | 0 (reference) |  |
|  | ≥ 12 | 13.94 (0.11) | -1.01 (-2.94 - 0.92) | 0.305 | -1.05 (-2.95 - 0.85) | 0.279 | -1.4 (-3.37 - 0.57) | 0.163 |
| Age at menopause | < 49 | 13.9 (0.16) | 0 (reference) |  | 0 (reference) |  | 0 (reference) |  |
|  | ≥ 49 | 13.98 (0.13) | 0.09 (-0.28 - 0.46) | 0.636 | 0.17 (-0.19 - 0.53) | 0.353 | 1.03 (0.35 - 1.71) | **0.003** |
| Interval from menarche to menopause | < 33 | 13.77 (0.18) | 0 (reference) |  | 0 (reference) |  | 0 (reference) |  |
|  | ≥ 33 | 14.03 (0.12) | 0.24 (-0.13 - 0.61) | 0.199 | 0.24 (-0.16 - 0.64) | 0.24 | 1.37 (0.59 - 2.16) | **0.001** |
| Duration of menarche until the study | < 50 | 14.03 (0.12) | 0 (reference) |  | 0 (reference) |  | 0 (reference) |  |
|  | ≥ 50 | 13.72 (0.17) | -0.57 (-1.16 - 0.02) | 0.056 | -0.57 (-1.2 - 0.06) | 0.075 | -1.08 (-2.07 - -0.08) | 0.034 |
| Duration after menopause | < 6 | 14.17 (0.16) | 0 (reference) |  | 0 (reference) |  | 0 (reference) |  |
|  | ≥ 6 | 13.82 (0.12) | -0.43 (-0.87 - 0.01) | 0.054 | -0.4 (-0.87 - 0.06) | 0.089 | -0.87 (-1.78 - 0.05) | 0.065 |

General linear models

Model 1: Adjusted for age

Model 2: Adjusted for age, diabetes mellitus, and systemic hypertension.

Model 3: Adjusted for age, diabetes mellitus, systemic hypertension, body mass index, triglycerides, and low-density lipoprotein cholesterol levels.

CI, confidence interval; IOP, intraocular pressure; SEM, standard error of the mean

All values represent aggregate estimates from survey-weighted analyses and do not contain individual-level participant data.
