# Supplementary material for: Association between female reproductive factors and intraocular pressure according to glaucoma status: A cross-sectional study of the Korea National Health and Nutrition Examination Survey
Source: PLoS One. 2026 Jul 29;21(7):e0353666. doi: 10.1371/journal.pone.0353666 (PMC13419174; doi:10.1371/journal.pone.0353666)
Supplement: S2 Table — (DOCX) [file pone.0353666.s002.docx]

**S2 Table.** Association between reproductive factors and intraocular pressure in participants without glaucoma after excluding those with bilateral oophorectomy.

| **Reproductive Factor** | **Category** | **Mean IOP (SEM)** | **Model 1** | | **Model 2** | | **Model 3** | |
| --- | --- | --- | --- | --- | --- | --- | --- | --- |
|  |  |  | **β (95% CI)** | **p-value** | **β (95% CI)** | **p-value** | **β (95% CI)** | **p-value** |
| Age at menarche | < 12 | 14.76 (0.96) | 0 (reference) |  | 0 (reference) |  | 0 (reference) |  |
|  | ≥ 12 | 13.88 (0.1) | -0.91 (-2.81 - 0.98) | 0.344 | -0.95 (-2.84 - 0.95) | 0.326 | -1.54 (-3.48 - 0.4) | 0.119 |
| Age at menopause | < 49 | 13.72 (0.14) | 0 (reference) |  | 0 (reference) |  | 0 (reference) |  |
|  | ≥ 49 | 13.99 (0.13) | 0.26 (-0.08 - 0.6) | 0.127 | 0.33 (-0.01 - 0.68) | **0.06** | 1.01 (0.37 - 1.66) | **0.002** |
| Interval from menarche to menopause | < 33 | 13.67 (0.15) | 0 (reference) |  | 0 (reference) |  | 0 (reference) |  |
|  | ≥ 33 | 14.02 (0.12) | 0.35 (0.02 - 0.68) | **0.037** | 0.34 (-0.01 - 0.69) | 0.054 | 1.14 (0.46 - 1.83) | **0.001** |
| Duration of menarche until the study | < 50 | 13.93 (0.11) | 0 (reference) |  | 0 (reference) |  | 0 (reference) |  |
|  | ≥ 50 | 13.73 (0.17) | -0.7 (-1.26 - -0.14) | **0.014** | -0.71 (-1.33 - -0.1) | **0.022** | -1.24 (-2.26 - -0.22) | **0.017** |
| Duration after menopause | < 6 | 14.1 (0.16) | 0 (reference) |  | 0 (reference) |  | 0 (reference) |  |
|  | ≥ 6 | 13.76 (0.11) | -0.59 (-1.02 - -0.17) | **0.007** | -0.57 (-1.01 - -0.13) | **0.011** | -1.03 (-1.81 - -0.25) | **0.01** |

General linear models

Model 1: Adjusted for age

Model 2: Adjusted for age, diabetes mellitus, and systemic hypertension.

Model 3: Adjusted for age, diabetes mellitus, systemic hypertension, body mass index, triglycerides, and low-density lipoprotein cholesterol levels.

CI, confidence interval; IOP, intraocular pressure; SEM, standard error of the mean

All values represent aggregate estimates from survey-weighted analyses and do not contain individual-level participant data.
