# Supplementary material for: Association between female reproductive factors and intraocular pressure according to glaucoma status: A cross-sectional study of the Korea National Health and Nutrition Examination Survey
Source: PLoS One. 2026 Jul 29;21(7):e0353666. doi: 10.1371/journal.pone.0353666 (PMC13419174; doi:10.1371/journal.pone.0353666)
Supplement: S3 Table — (DOCX) [file pone.0353666.s003.docx]

**S3 Table.** Association between reproductive factors and intraocular pressure in participants with glaucoma after excluding those with artificial menopause.

| **Reproductive Factor** | **Category** | **Mean IOP (SEM)** | **Model 1** | | **Model 2** | | **Model 3** | |
| --- | --- | --- | --- | --- | --- | --- | --- | --- |
|  |  |  | **β (95% CI)** | **p-value** | **β (95% CI)** | **p-value** | **β (95% CI)** | **p-value** |
| Age at menarche | < 16 | 14.77 (0.48) | 0 (reference) |  | 0 (reference) |  | 0 (reference) |  |
|  | ≥16 | 14.08 (0.51) | -0.71 (-1.89 - 0.46) | 0.234 | -0.95 (-2.05 - 0.16) | 0.092 | -0.84 (-2.05 - 0.38) | 0.175 |
| Age at menopause | < 48 | 14.69 (0.81) | 0 (reference) |  | 0 (reference) |  | 0 (reference) |  |
|  | ≥48 | 14.2 (0.39) | -0.1 (-1.61 - 1.42) | 0.898 | -0.3 (-1.83 - 1.23) | 0.699 | -1.35 (-2.55 - -0.14) | **0.029** |
| Interval from menarche to menopause | < 31 | 14.84 (0.81) | 0 (reference) |  | 0 (reference) |  | 0 (reference) |  |
|  | ≥31 | 14.15 (0.38) | -0.25 (-1.8 - 1.3) | 0.748 | 0.07 (-1.53 - 1.66) | 0.936 | -0.64 (-1.81 - 0.53) | 0.283 |
| Duration of menarche until the study | < 42 | 13.66 (0.57) | 0 (reference) |  | 0 (reference) |  | 0 (reference) |  |
|  | ≥42 | 14.73 (0.5) | -0.22 (-1.86 - 1.42) | 0.796 | 0.25 (-1.38 - 1.88) | 0.759 | 2.06 (-1.23 - 5.35) | 0.218 |
| Duration after menopause | < 6 | 13.67 (0.79) | 0 (reference) |  | 0 (reference) |  | 0 (reference) |  |
|  | ≥6 | 14.57 (0.47) | -0.44 (-2.44 - 1.55) | 0.662 | -0.02 (-1.99 - 1.95) | 0.982 | -2.43 (-6.52 - 1.66) | 0.242 |

General linear models

Model 1: Adjusted for age

Model 2: Adjusted for age, diabetes mellitus, and systemic hypertension.

Model 3: Adjusted for age, diabetes mellitus, systemic hypertension, body mass index, triglycerides, and low-density lipoprotein cholesterol levels.

CI, confidence interval; IOP, intraocular pressure; SEM, standard error of the mean

All values represent aggregate estimates from survey-weighted analyses and do not contain individual-level participant data.
