# Supplementary material for: Association between female reproductive factors and intraocular pressure according to glaucoma status: A cross-sectional study of the Korea National Health and Nutrition Examination Survey
Source: PLoS One. 2026 Jul 29;21(7):e0353666. doi: 10.1371/journal.pone.0353666 (PMC13419174; doi:10.1371/journal.pone.0353666)
Supplement: S4 Table — (DOCX) [file pone.0353666.s004.docx]

**S4 Table.** Association between reproductive factors and intraocular pressure in participants with glaucoma after excluding those with bilateral oophorectomy.

| **Reproductive Factor** | **Category** | **Mean IOP (SEM)** | **Model 1** | | **Model 2** | | **Model 3** | |
| --- | --- | --- | --- | --- | --- | --- | --- | --- |
|  |  |  | **β (95% CI)** | **p-value** | **β (95% CI)** | **p-value** | **β (95% CI)** | **p-value** |
| Age at menarche | < 16 | 14.88 (0.49) | 0 (reference) |  | 0 (reference) |  | 0 (reference) |  |
|  | ≥16 | 13.98 (0.49) | -0.94 (-2.16 - 0.27) | 0.127 | -1.17 (-2.36 - 0.01) | 0.052 | -1.68 (-3.5 - 0.14) | 0.07 |
| Age at menopause | < 48 | 14.75 (0.75) | 0 (reference) |  | 0 (reference) |  | 0 (reference) |  |
|  | ≥48 | 14.13 (0.38) | -0.43 (-1.9 - 1.05) | 0.571 | -0.58 (-2.06 - 0.9) | 0.439 | -2.1 (-3.7 - -0.49) | **0.011** |
| Interval from menarche to menopause | < 31 | 14.66 (0.75) | 0 (reference) |  | 0 (reference) |  | 0 (reference) |  |
|  | ≥31 | 14.18 (0.38) | -0.19 (-1.61 - 1.24) | 0.796 | 0.17 (-1.31 - 1.64) | 0.825 | -1.29 (-2.56 - -0.01) | **0.048** |
| Duration of menarche until the study | < 42 | 13.86 (0.57) | 0 (reference) |  | 0 (reference) |  | 0 (reference) |  |
|  | ≥42 | 14.59 (0.49) | -0.38 (-1.98 - 1.22) | 0.641 | 0.09 (-1.58 - 1.75) | 0.918 | 3.77 (0.25 - 7.3) | **0.036** |
| Duration after menopause | < 6 | 13.99 (0.82) | 0 (reference) |  | 0 (reference) |  | 0 (reference) |  |
|  | ≥6 | 14.43 (0.45) | -0.7 (-2.66 - 1.25) | 0.48 | -0.39 (-2.3 - 1.52) | 0.689 | 0.47 (-4.62 - 5.56) | 0.855 |

General linear models

Model 1: Adjusted for age

Model 2: Adjusted for age, diabetes mellitus, and systemic hypertension.

Model 3: Adjusted for age, diabetes mellitus, systemic hypertension, body mass index, triglycerides, and low-density lipoprotein cholesterol levels.

CI, confidence interval; IOP, intraocular pressure; SEM, standard error of the mean

All values represent aggregate estimates from survey-weighted analyses and do not contain individual-level participant data.
